# Angiome géant du foie

**DOI:** 10.11604/pamj.2014.19.174.5503

**Published:** 2014-10-20

**Authors:** Pierlesky Elion Ossibi, Karim Ibn Majdoub

**Affiliations:** 1 Service de Chirurgie Viscérale B, CHU Hassan II, Fès, Maroc

**Keywords:** Angiome, foie, tumeur benign, Angioma, liver, benign tumor

## Image en medicine

Les angiomes hépatiques sont des tumeurs bénignes du foie les plus fréquentes. Ils touchent tous les âges mais souvent des adultes avec une prédominance féminine. Souvent la lésion est unique de taille inférieure à 1 cm dans la moitié des cas. Elle peut être double ou multiple dans certains cas. Nous rapportons l'observation d'un patient de 40 ans sans antécédent notable qui présente depuis une semaine des douleurs isolées de l'hypochondre droit. L'examen clinique trouve un patient en bon état général avec une légère sensibilité de l'hypochondre droit. L’échographie abdominale est revenue en faveur d'un foie augmenté de taille de contours réguliers et d’échostructure hétérogène, siège d'une lésion nodulaire au niveau du segment VI et VII de 86 mm de grand axe. Le scanner abdominal montre un foie augmenté de taille de contours réguliers et de densité homogène, siège d'une volumineuse masse hypodense à centre liquidien, occupant tout le foie droit, mesurant 17 x 9 cm avec un rehaussement discontinu périphérique en motte au temps artériel (A) et un remplissage progressif et centripète aux temps portal et tardif (B).

**Figure 1:**
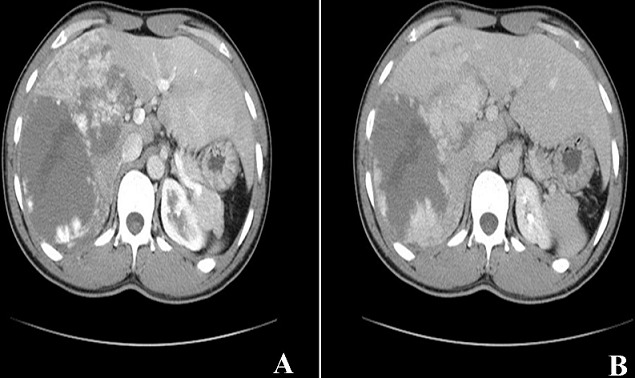
A) image sconnagraphique montrant le rehaussement discontinu périphérique en motte au temps artériel de la masse hépatique; B) image sconnagraphique montrant le remplissage progressif et centripète au temps portal de la masse hépatique

